# Imaging biomarkers in oncology: Basics and application to MRI

**DOI:** 10.1002/jmri.26058

**Published:** 2018-07-03

**Authors:** Isabel Dregely, Davide Prezzi, Christian Kelly‐Morland, Elisa Roccia, Radhouene Neji, Vicky Goh

**Affiliations:** ^1^ Biomedical Engineering, School of Biomedical Engineering & Imaging Sciences King's Health Partners, St Thomas' Hospital London, UK; ^2^ Cancer Imaging, School of Biomedical Engineering & Imaging Sciences King's College London, King's Health Partners, St Thomas' Hospital, London UK; ^3^ Radiology Guy's & St Thomas' NHS Foundation Trust London UK; ^4^ MR Research Collaborations Siemens Healthcare Frimley UK

**Keywords:** magnetic resonance imaging, imaging biomarkers, oncology, precision medicine

## Abstract

Cancer remains a global killer alongside cardiovascular disease. A better understanding of cancer biology has transformed its management with an increasing emphasis on a personalized approach, so‐called “precision cancer medicine.” Imaging has a key role to play in the management of cancer patients. Imaging biomarkers that objectively inform on tumor biology, the tumor environment, and tumor changes in response to an intervention complement genomic and molecular diagnostics. In this review we describe the key principles for imaging biomarker development and discuss the current status with respect to magnetic resonance imaging (MRI).

**Level of Evidence:**

5

**Technical Efficacy:**

Stage 5 J. Magn. Reson. Imaging 2018;48:13–26.

Cancer affects 14.1 million new patients yearly and is the second most common killer disease worldwide.^1^ Clinicians have long recognized that cancer represents a very heterogeneous disease. Patients with the same clinical presentation, tumor type, and stage may respond very differently to the same therapies and have different oncological outcomes. A better understanding of the extent of the genomic and molecular heterogeneity within cancers, as demonstrated in renal cell cancer,^2^ has led to a refocusing of clinical management in recent years from a global to a more targeted approach.^3^ Currently, cancer therapies aim to be personalized to the patient's cancer, either to cure where there is limited disease, or to extend progression‐free survival (PFS) where disease is advanced, yet maintaining a good quality of life, so‐called “precision cancer medicine.”

The US Food and Administration (FDA) approval of bevacizumab in 2004 for first‐line metastatic colorectal cancer, after a Phase III trial demonstrated an improvement in median PFS of 4 months,^4^ has paved the way for an increasing number of licensed molecular targeted therapies. These include targeted HER‐2 (human epidermal growth factor receptor 2) therapy (trastuzumab) for HER‐2 overexpressing breast cancer and gastric/gastroesophageal cancer; targeted EGFR (epidermal growth factor receptor) therapy (cetuximab) for RAS wildtype colorectal cancer; targeted EGFR therapy (gefitinib or erlotinib) for EGFR mutated nonsmall‐cell lung cancer; crizotinib for ALK (anaplastic lymphoma kinase) gene rearrangement nonsmall‐cell lung cancer (present in ∼5% of adenocarcinomas); and multikinase inhibitors (pazopanib, sorafenib, sunitinib) or mammalian target of rapamycin (mTOR) inhibitors (everolimus) for advanced renal cell cancer.

Trials of these therapies have highlighted the need for better diagnostics to support patient stratification for therapy as well as a rethink of how we gather evidence for novel therapeutics that may only work for a subgroup of patients. There has been burgeoning development of precision diagnostics as a consequence. For single agents targeted to clearly defined genetic “driver” alterations, companion diagnostics improve the selection of patients for therapy, eg, HER‐2 expression to guide trastuzumab therapy and O^6^‐methylguanine‐DNA‐methyltransferase (MGMT) methylation to guide temozolomide therapy. There has also been increasing interest in genomic analysis to guide therapy with the move from single to multiagent regimens and also to improve prognostication, eg, oncotype DX in breast cancer that predicts the likelihood of recurrence from a 21‐gene signature as well as the likelihood of response to chemotherapy.

While the advantages of genomic analysis and molecular analysis to improve patient stratification and to assist drug development is clear, in practice there have been continuing challenges to implementation. Some putative biomarkers may be invalid, as shown with EGFR expression for cetuximab.^5^ Cancers are also temporally and spatially heterogeneous, ie, a biopsy or assay may only reflect a moment in time, or one of a number of lesions. This plasticity has been a reason for mixed responses to therapies and the development of therapy resistance during previously effective targeted therapy.^6^ There may also be issues such as suboptimal methodology, challenging assays, validation, regulatory issues, and governance or cost that are a challenge for multicenter clinical trials.

Imaging still has an important role to play in personalized cancer medicine.^7^ Imaging is performed widely for the detection and characterization of cancer, for staging, for monitoring therapy, for detecting disease recurrence, or surveillance; imaging biomarkers hold great potential for optimizing patient care. The role of magnetic resonance imaging (MRI) has evolved within oncological practice in recent years. Previously reserved as an adjunctive problem‐solving tool, the primary use of MRI has increased, such that MRI is now the primary imaging assessment tool for many cancers and plays an important part in management decisions. It is the initial imaging modality for diagnosing prostate cancer and myeloma; for staging rectal, cervical, and endometrial cancer; and for response assessment in hepatocellular cancer. In this review we will describe what constitutes an imaging biomarker, the principles of imaging biomarker development, and the current status of imaging biomarkers with respect to MRI.

## What Constitutes a Biomarker?

The term “biomarker” refers to a characteristic that is measured objectively, as an indicator of normal biological processes, pathological changes, or response to an intervention.^8^ It includes molecular, histologic, radiographic, or physiologic characteristics. In terms of imaging, this may include anatomical, functional, and molecular characteristics.^7^ The advantages of imaging are its versatility, its widespread use, its relatively noninvasive nature (facilitating whole body imaging as well as longitudinal studies in individuals, thus capturing spatial and temporal heterogeneity), and its inherently quantitative nature. Imaging biomarkers may reflect a general cancer hallmark, eg, proliferation, metabolism, angiogenesis, apoptosis; specific molecular interactions; or agnostic features.^9^ Imaging biomarkers in cancer patients include biomarkers for detection (the identification of disease), prediction (the prediction of risk of disease or therapeutic outcome), prognostication (the prediction of oncological outcome), and response assessment (the evaluation of change with therapy). A number of imaging biomarkers are well established in clinical practice. Examples include staging with the American Joint Committee on Cancer (AJCC) TNM (tumor, node, metastasis) staging system (a prognostic biomarker) and objective response assessment by RECIST (Response Evaluation in Solid Tumors)^10^ in clinical trials (a response biomarker).

## Imaging Biomarkers: From Discovery to Clinical Practice

**Figure 1:**
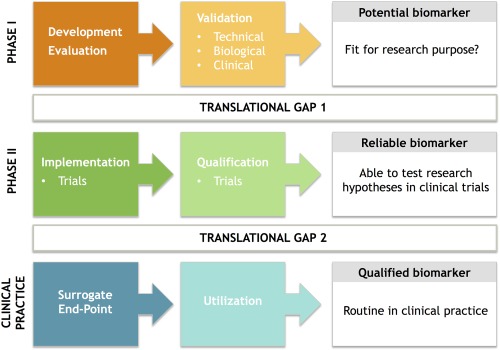
Schema highlighting steps taken in developing a potential imaging biomarker

For new potential imaging biomarkers several steps, often in parallel and complementary to each other, need to be undertaken for translation into clinical practice. These can be divided into the following phases following discovery: development and evaluation, validation, implementation, qualification, and utilization, essentially crossing two main translational gaps, translation into patients and translation into practice (Fig. 1).

In the initial phase, including development, evaluation, and validation, the aim is to ensure that the potential biomarker is robust and fit for purpose. Technical validation includes assessment of accuracy, precision, repeatability, and reproducibility across single and multiple centers; biological and clinical validation ensure that the biomarkers are linked to tumor biology, outcome variables, and thus of actual value in guiding decision‐making in patients. During this phase, initial health economic analysis may also be undertaken to identify if there are cost barriers to implementation. Once the biomarker is established, it should be reliable enough to be implemented in clinical trials to test research hypotheses.

During the next phase, qualification of the biomarker may also be undertaken in large prospective trials. Qualification aims to confirm that the biomarker is associated with the clinical endpoint of interest and aims to demonstrate cost effectiveness and health impact. This supportive evidence is key to the translation into clinical practice and widespread utilization. Key recommendations have been proposed in a recent consensus article.^11^

## Advantages of MRI as an Imaging Biomarker

Ideally, there are a number of characteristics an imaging biomarker should have (Table 1). MRI has many advantages, including its superior soft‐tissue contrast, high spatial resolution; its ability to obtain multiple contrasts in a single examination; and its ability to assess physiology, eg, vascularization, oxygenation, and diffusion. Assessment of the molecular environment is also achievable, albeit at a lower sensitivity compared to positron emission tomography (PET). A number of MRI biomarkers are already established or well on their way to being established in clinical practice for oncological assessments (Table 2). These include BI‐RADS (Breast Imaging Reporting and Data System),^12^ LI‐RADS (Liver Imaging Reporting and Data System),^13^, ^14^ and PI‐RADS (Prostate Imaging Reporting and Data System)^15^ for the diagnosis of breast, hepatocellular cancers, and prostate, respectively, in addition to TNM staging and RECIST response evaluation. Quantitative biomarkers that have crossed the first translational gap and are being used to test hypotheses in research studies and clinical trials include vascular parameters such as initial area under the gadolinium curve (iAUGC) or transfer constant (K^trans^) from dynamic gadolinium enhanced (DCE) contrast imaging and apparent diffusion coefficient (ADC) from diffusion‐weighted MRI (Table 2).

**Table 1:** Key Characteristics and Challenges for MRI Biomarkers

Characteristics	Challenges for MRI	Developments
Sensitive	Signal to noise ratio (SNR) Contrast to noise ratio (CNR) Spatial resolution Artifacts	New sequences
Specific & biologically relevant	Targeted versus physiological or morphological imaging	Evaluation of more targeted imaging, eg, receptor imaging, targeted nanoparticles
Robust	Variance among imaging systems, manufacturers & practice	Multivendor & multicenter involvement to standardize data acquisition, reconstruction & analysis
Quantifiable & reproducible	Variance among imaging systems, manufacturers & practice	Advanced acquisition and reconstruction to exploit data redundancy Single‐sequence MRI to acquire several image contrasts in a coregistered fashion, eg, MR fingerprinting
Cost effective	Higher cost compared to computed tomography (CT) or ultrasound (US)	Reduction in scanner time with faster acquisitions

**Table 2:** Established and Validated MRI Biomarkers in Clinical Use

Biomarker	Characteristic	MRI sequence
Established biomarkers in clinical practice		
Detection & characterization		
BI‐RADS (Breast Imaging Reporting and Data System) PI‐RADS (Prostate Imaging Reporting and Data System) LI‐RADS (Liver Imaging Reporting and Data System)	Lesion morphology	T2‐weighted, T1‐weighted, diffusion weighted, postcontrast‐enhanced imaging
Curve shape	Degree of vascularization	Dynamic T1‐weighted imaging following intravenous injection of gadolinium‐based contrast agent
Staging		
TNM stage	Tumor morphology, presence of nodes, and metastases	T2‐weighted, T1‐weighted imaging ± diffusion weighted, postcontrast‐enhanced imaging
Response		
RECIST (Response Evaluation Criteria In Solid Tumors)	Change in tumor size	T2‐weighted imaging
Validated biomarkers in clinical cancer research		
Apparent diffusion coefficient (ADC)	Cellularity	Diffusion‐weighted imaging, at least 2 b‐values
Initial area under the gadolinium curve (iAUGC) Transfer constant (*K* ^*trans*^)	Perfusion Permeability	Dynamic T1‐weighted imaging following intravenous injection of gadolinium‐based contrast agent

## Morphology‐Based MRI Biomarkers

Current morphology‐based cancer biomarkers utilize the multiple contrasts and high spatial resolution of MRI. *T*_2_‐weighted and *T*_1_‐weighted sequences are part of every cancer protocol. *T*_2_‐weighting highlights structures with a longer *T*_2_ relaxation time. Thus, organs with a high water content, eg, bladder, appear of high signal on *T*_2_‐weighted imaging, while cancers typically appear of intermediate signal. *T*_2_‐weighted image contrast is encoded by a long echo time (TE) and long repetition time (TR). Typically, 2D imaging is performed in axial, sagittal, and/or coronal planes using a fast/turbo spin echo sequence. 3D imaging can be performed using a 3D *T*_2_w‐TSE with optimized flip angle evolution along the echo train (eg, Siemens SPACE, Philips VISTA, GE CUBE). *T*_1_‐weighting highlights structures with a short *T*_1_, eg, fat, melanin. *T*_1_‐weighted image contrast is encoded by a short TE and short TR. *T*_1_w‐MRI is acquired with fast gradient echo sequences in 2D (Siemens FLASH, Phillips FFE, GE GRE) or 3D (Siemens VIBE, Philips THRIVE, GE Lava).

### Diagnostic Biomarker

**Figure 2:**
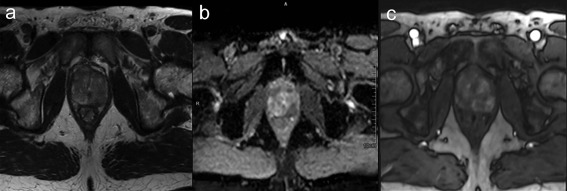
Multiparametric prostate MRI demonstrates a left mid‐gland PI‐RADS 5 peripheral zone lesion extending beyond the prostate **(a:** T_2_‐weighted, **b:** diffusion‐weighted apparent diffusion coefficient map, **c:** arterial phase dynamic contrast‐enhanced T_1_‐weighted image).

A key example of a recently established diagnostic biomarker is PI‐RADS in suspected prostate cancer, currently on version 2.0,^15^ utilizing multiparametric MRI. The PROMIS trial^16^, ^17^ has recently published its findings confirming a role for multiparametric MRI in the diagnostic pathway of patients with suspected prostate cancer. This enrolled 740 men, 576 of whom underwent 1·5T multiparametric MRI followed by both transrectal ultrasound (TRUS) biopsy and template prostate mapping biopsy. On template prostate mapping biopsy, 408 (71%) of 576 men had cancer with 230 (40%); of 576 patients it was clinically significant. For clinically significant cancer, multiparametric MRI was more sensitive (93%, 95% confidence interval [CI] 88–96%) than TRUS biopsy (48%, 42–55%; *P* < 0·0001). Using multiparametric MRI to triage men might allow 27% of patients to avoid a primary biopsy and improve detection of clinically significant cancer. Using a structured reporting scheme such as PI‐RADS standardizes practice, provides an objective score of the likelihood of disease, and helps direct targeted biopsy. Risk scores to assess the likelihood of clinically significant cancer are defined as PI‐RADS 1: very low, PI‐RADS 2: low, PI‐RADS 3: intermediate, PI‐RADS 4: high, to PI‐RADS 5: very high. A meta‐analysis has revealed overall high sensitivity and specificity of 0.74 and 0.88, respectively, for prostate cancer detection with PI‐RADS.^18^, ^19^ MRI is performed with a multiparametric acquisition of at least *T*_2_‐weighted and diffusion‐weighted sequences^20^ (Fig. 2). This combines high resolution, high soft‐tissue contrast of *T*_2_‐weighted imaging with the diffusion‐weighted imaging sensitivity for cancer.^21^ Additional dynamic contrast‐enhanced sequences provide information of wash‐in and wash‐out characteristics and may provide additional diagnostic value. A recent study has demonstrated an increase in the probability of cancer detection of 16%, 16%, and 9% for PI‐RADS category 2, 3, and 4 lesions, respectively, with DCE‐MRI.^22^

### Prognostic Biomarker: Staging

**Figure 3:**
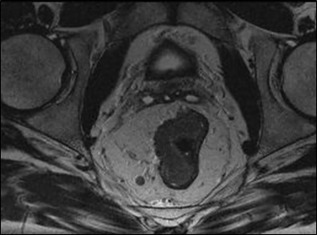
T_2_‐weighted axial image demonstrates a T3N1 rectal cancer extending beyond the rectal wall but not involving the potential resection margin

Staging is an important imaging biomarker for patient stratification. MRI is the primary staging modality for a number of cancers including rectal cancer. In addition to TNM‐Stage grouping, which provides an indication of relative 5‐year overall survival (Stage I [localized, T1/2], node negative: 95%; vs. Stage IV [metastatic, any T,N]: 11%), MRI also has a predictive role in terms of likely involvement of the resection margin and PFS^23^, ^24^, ^25^ (Fig. 3).

### Response Biomarker: RECIST

RECIST criteria provide a standardized, objective assessment of response to therapy in clinical trials.^10^ Classification of response is divided into four categories (complete response, partial response, stable disease, progressive disease) based on size change of specified measurable target lesions (>1 cm) or nodes (>1.5 cm short axis) (Table 3). From a regulatory perspective, RECIST remains the key response biomarker in clinical trials and is used as a surrogate endpoint.

**Table 3:** Response Categorization Based on Changes in Target and Nontarget Lesions

RECIST		
Categorization	Target lesions	Nontarget lesions
Complete response (CR)	Disappearance of all target lesions (TL). All nodes <10 mm, ie, nonpathological	Disappearance of all nontarget lesions. All nodes <10 mm, ie, nonpathological
Partial response (PR)Stable disease (SD)	>30% decrease in the sum of TL diameters Neither PR nor PD	Non CR/PD: Persistence of ≥1 nontarget lesion
Progressive disease (PD)	>20% increase in the sum of TL diameters. Absolute increase of at least 5 mm. New lesions	Unequivocal progression of existing nontarget lesions New lesions

Target lesions: Up to 5 measured, 2 maximum per organ.

## Validated MRI Biomarkers Requiring Qualification

### Diffusion‐Weighted MRI

ADC is a biomarker that has crossed the first translational gap and is used to test research hypotheses in clinical trials.^26^ The biophysical basis of diffusion‐weighted imaging is the microscopic displacement of water molecules (Δx ≊ 30 μm in Δt = 50 msec) due to thermal Brownian motion. In cancers the tumor environment restricts this motion, thus a measurement of the effective displacement, the ADC, gives important microscopic information. Tumor ADC from b‐values less than 1000 s/mm^2^ effectively provide a measure of the extracellular space; although cell size, cell arrangements, cell density, integrity of cell membranes, glandular structures, extracellular space viscosity, and tortuosity will influence this measurement. Studies have correlated ADC with histological grade in a number of cancers.^27^, ^28^, ^29^, ^30^

The diffusion image contrast is encoded by using a gradient pair (Stejskal‐Tanner gradient^26^), which can be either a bipolar gradient pair in gradient echo or the same polarity in spin echo. This gradient causes a change in the resonant Larmor frequency of a spin isochromat, leading to the following phase accumulation φ:

$$\phi =\;\overset{t}{\underset{0}{\int }}\Delta \omega dt^{\prime }=\;\gamma \overset{t}{\underset{0}{\int }}\vec{G}\left(t^{\prime }\right)\cdot \vec{r}(t^{\prime })dt^{\prime }$$

where $\vec{G}$ is the applied gradient waveform applied for a duration *t*, $\;\vec{r\;}\;$ is the spatial position of the spin isochromat, and γ the gyromagnetic ratio. Thus, spins, which move during the application of the gradient pair, will not be properly rephased. This loss in phase coherence secondary to spatial displacement causes a reduction in the signal. For random spin diffusion motion in an image voxel, this signal cancellation is related to the variance of the Gaussian phase distribution <ϕ^2^> and the product *bD*:

$$S=S_{0}e^{-\langle \phi^{2}\rangle }=S_{0}e^{-bD}$$

Where S is the diffusion‐weighted signal and *S*_0_ is the signal without diffusion weighting.

**Figure 4:**
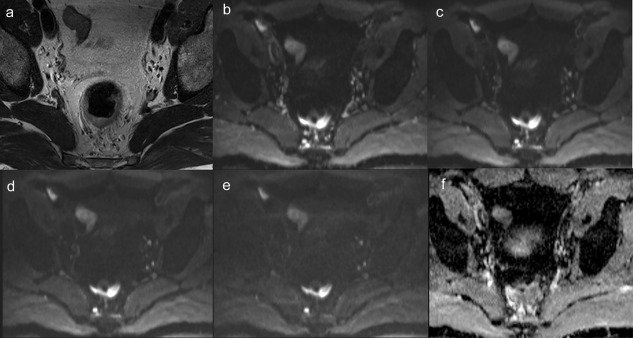
The T_2_ axial oblique image **(a)** of a rectal cancer, diffusion‐weighted images with increasing b‐weighting 0 **(b)**, 100 **(c)**, 500 **(d),** and 800 s/mm^2^ **(e),** and corresponding ADC_0‐800_ map **(f)** is shown. Signal loss is demonstrated within the rectal cancer with increasing b‐weighting. The signal loss is greater for normal tissue than for the cancer.

Thus, the degree of attenuation depends on the dimensionless product of the diffusion coefficient D (in mm^2^/sec) and the b‐value (in sec/mm^2^). The b‐value is used to control the diffusion‐weighted contrast with higher diffusion weighting at higher b‐values. Typically, b‐values of 0–1500 s/mm^2^ are applied in clinical practice and ADC is obtained from monoexponential fitting of the signal loss (Fig. 4). In practice, other factors contribute to signal loss including *T*_2_‐relaxation and bulk motion. In a given voxel, ADC will reflect the relative contribution of the different compartments.

A number of studies have evaluated ADC as a response biomarker in a number of tumor types across different therapies in research studies including the multicenter setting. These studies have shown that a common pattern is an increase in ADC_mean_ to a varying extent with different therapies. This may occur within days of starting treatment; a higher change in ADC_mean_ is also associated with a pathological good response.^31^, ^32^, ^33^, ^34^, ^35^, ^36^, ^37^, ^38^, ^39^

The variability of ADC in clinical studies has been reported to be relatively low at ≤15%^40^ and in ice‐water phantom studies as low as 3%.^41^ Nevertheless, there are considerations to be made in the trial setting^42^ and technical challenges to acquiring robust diffusion‐weighted biomarkers and qualification as a biomarker.^26^ TR should be sufficiently long to avoid underestimation of ADC due to *T*_1_ saturation effect; TE should be minimized to achieve better signal‐to‐noise ratio (SNR), to minimize motion and susceptibility artifacts. Good fat suppression is required to minimize ghosting artifacts; short tau inversion recovery (STIR) may be preferred to spectral presaturation attenuated by inversion recovery (SPAIR) or chemical shift selective water‐only excitation techniques, where a large field of view is necessary at 1.5T, as STIR is less sensitive to B0 field inhomogeneities. Geometric distortion and susceptibility artifacts caused by eddy currents related to EPI may be improved by shortening the echo train length, eg, through adapting the receiver bandwidth to reduce the echo spacing, use of parallel imaging, zoomed excitation, or readout segmented imaging.

### Dynamic Contrast‐Enhanced MRI

DCE MRI refers to the rapid acquisition of a time series of *T*_1_w images before, during, and after intravenous administration of a gadolinium‐based contrast agent. Gadolinium contrast agents are small hydrophilic molecules with a short circulation half‐life, typically <1 hour. These contrast agents shorten the *T*_1_‐relaxation rate, and thus cause signal enhancement related to the delivery and leakage rate of contrast agent within the tissue of interest, providing a surrogate measure of angiogenesis.

While qualitative assessment of curve shape is an established imaging biomarker, eg, for the evaluation of suspected breast and prostate cancer, the use of quantitative vascular parameters remains in the domain of clinical trials. In terms of qualitative assessment, three distinct curve shapes are recognized: Type 1) slow rising enhancement (benign); Type 2) rapid enhancement with a plateau (may be malignant); and Type 3) rapid enhancement followed by rapid washout (malignant).

For assessing quantitative parameters, baseline *T*_1_ mapping is required usually with a dual flip angle 3D *T*_1_‐weighted spoiled gradient recalled echo acquisition (e.g., 2°/18°) with other parameters remaining constant.

The baseline *T*_1_ value (*T*_10_) is estimated from fitting the signal intensity of the images acquired with different flip angles to the following equation:

$$S=\frac{S_{0}\left(1-E_{1}\right)\sin\left(\alpha \right)}{1-E_{1}\cos\left(\alpha \right)}$$

where S is acquired *T*_1_‐weighted signal, α represents the applied flip angle in each acquisition, *S*_0_ is the *T*_1_ fully relaxed signal, and $E_{1}=e^{\frac{-TR}{T_{10}}}$, where TR is the sequence repetition time. Contrast agent administration, typically 0.1 mmol/kg body weight, is followed by a dynamic acquisition for up to 5 minutes with a temporal resolution on the order of 3–5 seconds between acquisitions. Contrast agent concentration may be estimated with the following equation:

$$\frac{1}{T1\left(t\right)}=\frac{1}{T_{10}}+r_{1}C$$

where *T*_1_(t) represents the *T*_1_ change over time due to the contrast agent, *T*_10_ represents the *T*_1_ of the tissue at baseline, *r*_1_ represents the *T*_1_ relaxivity of the contrast agent, and C represents the unknown contrast concentration.

The Tofts and Kermode model^43^ is applied most commonly to determine *K* ^*trans*^ (a product of flow and transfer permeability):

$$\frac{dC_{t}(t)}{dt}=K^{trans}C_{p}(t)-k_{ep}C_{t}(t)$$

where *C_t_(t)* and *C_p_(t)* represent the contrast agent concentration in tissue and plasma as a function of time, respectively, *K* ^*trans*^ represents transfer constant, *k*_*ep*_ represents the rate constant; or as an extended model to account for the contrast agent in the vasculature, when vascular volume cannot be neglected.

$$C_{t}\left(t\right)=v_{p}C_{p}\left(t\right)+\;K^{trans}\overset{t}{\underset{0}{\int }}C_{p\;}\left(t^{\prime }\right)\exp\left(\frac{-K^{trans}\left(t-t^{\prime }\right)}{v_{e}}\right)dt^{\prime }$$

where *C_t_(t)* and *C_p_(t)* represent the contrast agent concentration in tissue and plasma, respectively, *K* ^*trans*^ represents transfer constant, *k*_*ep*_ represents the rate constant; *v*_*p*_ represent the fractional plasma volume; and *v*_*e*_ the fractional extracellular extravascular volume.

**Figure 5:**
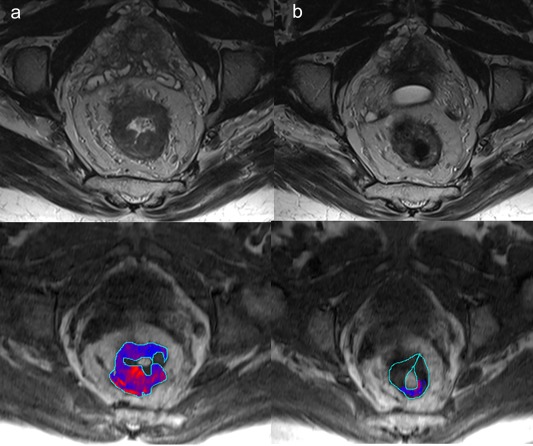
T_2_‐weighted **(a)** and corresponding transfer constant maps (*K* ^*trans*^, **b)** before and after three cycles of therapy with an antiangiogenic and triplet chemotherapy. A decrease in tumor vascularization is noted following three cycles of therapy.

In the last 15 years, over 110 studies in 2268 patients have utilized quantitative DCE‐MRI as a biomarker in clinical studies and trials reflecting the use of DCE‐MRI to assess vascular activity in drug development,^44^ in particular to assess the effect of antiangiogenic or antivascular therapy (Fig. 5). Consistent reduction in the initial area under the gadolinium curve (iAUGC) and *K* ^*trans*^ have been found for a number of therapies including VEGF‐targeted agents (bevacizumab) and multikinase inhibitors (pazopanib, sunitinib, sorafenib), as early as a few hours after dosing.

Nevertheless, the variability of *K* ^*trans*^ in clinical studies remains a major issue (>50%), and baseline reproducibility has been utilized in clinical trials on an individual basis in order to be able to determine whether the measured change is related to therapeutic effect. Accurate determination of the arterial input function (AIF), which characterizes contrast agent arrival in a feeding blood vessel within the tumor, remains a challenge to accurate quantification. As an alternative to subject‐specific direct measurement of AIF (subject to flow artifacts, nonlinear effects of high contrast agent concentrations, and partial volume effects), population‐based AIFs^45^ or reference tissue‐based methods^46^ have been advocated. Accurate *T*_1_‐mapping also remains a challenge, as B1 inhomogeneity, particularly at 3T and higher field strengths, limit the accuracy of *T*_1_‐estimates derived from the typically employed variable flip angle technique. Recent developments propose to include B1+ for *T*_1_‐mapping.^47^ To overcome the challenge of achieving both high spatial and temporal resolution for the DCE data acquisition, advanced methods have been proposed, such as combining parallel imaging, compressed sensing and non‐Cartesian sampling,^48^ view sharing,^49^ and motion compensation.^50^

## Emerging MRI Biomarkers

Further emerging quantitative biomarkers are undergoing evaluation (Table 4), related to the following techniques: intravoxel incoherent motion (IVIM), diffusion kurtosis imaging, blood and tissue oxygenation level‐dependent MRI (BOLD/TOLD), MR elastography, and relaxometry imaging. There has also been growing interest in extracting additional agnostic features from standard and quantitative MRI sequences, so‐called radiomics.^9^

**Table 4:** Emerging Biomarkers Undergoing Validation in Research Studies

Emerging biomarkers	Measure/biological correlate	MRI sequence
*f*, D*	Pseudoperfusion	Multiple low b‐value diffusion weighted imaging (intravoxel incoherent motion, IVIM)
Kurtosis (K_app_)	Microstructural complexity	Diffusion kurtosis imaging (DKI)
R2* R1 ΔR2* ΔR1	Relaxation rate Oxygenation	Blood oxygenation level dependent imaging (BOLD) Tissue oxygenation level dependent imaging (TOLD) ± oxygen/carbogen challenge
Elasticity Viscosity	Tissue mechanics and viscoelastic parameters	Elastography: motion sensitive sequence to encode shear wave propagation
Specific metabolites, eg, Choline	Metabolite concentration	Spectroscopy
T1 T2	Relaxation time Microenvironment	Multiecho relaxometry imaging
Texture features	Heterogeneity	Any

### Pseudodiffusion and Intravoxel Incoherent Motion

Bulk water motion in capillaries can also cause phase dispersion in diffusion‐weighted MRI.^51^, ^52^ The loss in signal is similar to that seen with true diffusion and more marked at low b‐values. Diffusion‐weighted MRI always measures both, but the relative contribution depends on the choice of b‐values. The contribution of true diffusion and perfusion towards signal loss can be defined as follows:

$$S\left(b\right)=\left(1-f_{v}\right)e^{-bD}+f_{v}e^{-bD^{*}}$$

where *S* is the acquired diffusion‐weighted signal, *b* represents the b‐value, *f*_v_ represents the fractional volume of flowing water molecules within capillaries; (1−*f*_v_) is the fraction of molecules undergoing true diffusion; D represents tissue diffusion coefficient and D* the pseudodiffusion coefficient. D* the pseudodiffusion coefficient associated with blood flow is about 10 × 10^−3^ mm^2^/sec in the brain and 70 × 10^−3^ mm^2^/sec in the liver compared to D, which is 1 × 10^−3^ mm^2^/sec.

Assessing *f*_*v*_ and D* may be feasible for patients with poor renal function, an allergy precluding intravenous administration of contrast agent, or at high risk of developing nephrogenic systemic fibrosis.^53^

However, one of the issues highlighted to date is the poor test–retest variability of *f* and D*,^54^ on the order of >100% in some cancers, eg, rectal.^55^ There also appears some contention as to technical/biological correlates: while some studies have shown a relationship between IVIM and DCE‐MRI parameters,^56^, ^57^, ^58^ others have not in some cancers, eg, hepatocellular carcinoma.^59^ One also has to be aware that flow from glandular secretions, eg, pancreas, may be difficult to separate from micro‐capillary perfusion. A potential application is as a diagnostic biomarker, where current characterization may be a challenge, eg, pancreas.^60^, ^61^

### Apparent Diffusional Kurtosis

Diffusion kurtosis imaging characterizes non‐Gaussian diffusion behavior at high b‐values ranging from 1000–3000 sec/mm^2^. A polynomial decay model is fitted to an acquisition using at least three b‐values to obtain *D*_*app*_ and *K*_*app*_ representing the heterogeneity of the cellular microstructure. The diffusion signal *S*_i_ for a given b‐value b_I_ is given by_:_

$$S_{i}=S_{0}*e^{b_{I}D_{app}+\frac{1}{6}Db_{I}^{2}D^{2}{\;}_{app}K_{app}}$$

where *S*_0_ is the signal without diffusion weighting, *K*_*app*_ is the apparent diffusional kurtosis, and *D*_*app*_ is the diffusion coefficient. *K*_*app*_ reflects the signal curvature away from a monoexponential fit. The rationale proposed for assessing kurtosis is that it may better reflect the tumor intracellular microstructure,^62^, ^63^ although it will also be influenced by extracellular properties. Higher kurtosis may be noted where there are higher intracellular interfaces; for example, increased nuclear‐cytoplasmic ratio of tumor cells.^64^ Preliminary studies in prostate cancer have suggested potential as a diagnostic biomarker,^65^ eg, to improve characterization (grading),^66^, ^67^ although not all studies have confirmed additional advantages over monoexponential ADC.^68^, ^69^ Studies have also suggested its potential as a response biomarker. A study in hepatocellular carcinoma has suggested that K_app_ performs better than ADC in detecting viable disease posttreatment.^70^

### Tumor Elasticity and Viscosity

Magnetic resonance elastography (MRE) quantifies the viscoelastic properties of tissue by assessing its elastic response to an applied force, similar to palpation in clinical practice. The applied force consists of harmonic mechanical waves, ranging typically between 20 and 80 Hz in frequency and propagated into the human body by a vibrating transducer applied to the body surface. The consequent tissue motion is captured using rapid motion‐sensitive MRI sequences. Through mathematical inversion algorithms, the local shear wave properties can be derived from the periodical variations in MRI signal; the local viscoelastic parameters (elasticity and viscosity) are then calculated using the complex shear modulus equation.^71^ The underpinning experimental observation for the application of MRE to cancer is that malignancy increases stiffness through collagen deposition in the extracellular matrix and raises interstitial pressure levels from its abnormal vasculature.^72^ MRE has shown promising potential for the characterization of focal lesions (benign vs. malignant) in multiple organs, including the liver,^73^ breast,^74^ pancreas,^75^ and kidney.^76^ It may also serve as a potential biomarker of treatment resistance.

### Tumor Oxygenation

Tumor oxygenation may be measured indirectly by BOLD and TOLD‐MRI techniques. With BOLD MRI, endogenous hemoglobin acts as a paramagnetic contrast agent that increases the transverse relaxation rate (R2*) in blood and surrounding tissue. R2* is measured from multiple spoiled gradient recalled echo images with increasing echo times. R2* is calculated from the gradient of a straight line fitted to a plot of ln‐signal intensity to TE. Higher R2* reflects higher deoxyhemoglobin levels and lower blood oxygenation. R2* may have a role as a response biomarker. One study has shown that R2* is inversely correlated to blood volume and increases in breast cancer treated with two cycles of neoadjuvant chemotherapy with greater changes in patients with pathological response.^77^ However, BOLD measurements will be affected by the underlying tissue relaxivity and will be affected by hemorrhage and susceptibility artifacts.

With TOLD MRI the longitudinal relaxation rate (R1) is measured. R1 is sensitive to changes in the O_2_ dissolved in blood plasma and interstitial fluid. When a hyperoxic gas is inhaled, the excess oxygen dissolved will result in a higher R1 value. A positive change in R1 will identify areas with fully saturated hemoglobin. Areas where there is no positive change in R1 may reflect regions of hypoxia, particularly if perfusion is present. Current approaches are focusing on the feasibility of combining R2* and R1 measurement with oxygen challenge to assess tumor oxygenation.^78^

### Quantitative MRI With or Without Exogenous Contrast agents

In current clinical practice, a diagnosis based on MRI primarily relies on the *qualitative* assessment of images. In contrast, *quantitative* measurements of tissue properties with or without endogenous contrast agents may provide more accurate and reproducible information. Without the use of exogenous contrast agents, relaxometry yields quantitative measurement of intrinsic tissue relaxation times *T*_1_ and *T*_2_,^79^, ^80^, ^81^, ^82^ *T*_2_*, proton density. In addition, important molecular information about tumor physiology and metabolism (“tumor microenvironment”) may be obtained from MR spectroscopy (MRS),^83^, ^84^, ^85^, ^86^, ^87^, ^88^ chemical exchange saturation transfer imaging (CEST),^89^ and amide proton transfer (APT).^90^ Further, relaxometry with exogenous contrast agents enables imaging of perfusion, using either gadolinium‐based contrast agents^91^ and dynamic *T*_1_w (DCE), as discussed previously, or *T*_2_*w MRI (dynamic susceptibility contrast‐enhanced [DSC]). Superparametric iron oxide (SPIO) nanoparticles in combination with *T*_2_w and *T*_2_*w MRI have been developed as imaging probes for targeted molecular MRI, cell tracking, and drug delivery (“theranostics”).^92^, ^93^, ^94^ Alternatively, highly specific, background‐free imaging can be achieved via nonproton imaging using, eg, F‐19^95^, ^96^, ^97^ or hyperpolarized agents C‐13.^98^, ^99^ However, these require hardware modifications to be able to image the nonproton frequencies.

Novel quantitative methods have also been proposed to acquire several tissue properties at once.^100^, ^101^ A method termed “MR‐fingerprinting” utilizes a (pseudo) randomized acquisition sequence to encode a tissue‐specific “Fingerprint” into an MR time series signal.^102^ This has recently also been adapted and applied to cancer imaging.^103^, ^104^, ^105^

Finally, to achieve its full potential, a key challenge of multiparametric MRI is standardization across multiple platforms, which involves the use of phantoms and careful review of implementation.^106^

### Radiomics

Radiomics is an evolving area in medical imaging whereby a large number of features are extracted and interpreted using bioinformatic approaches.^9^, ^107^ The underlying rationale for radiomics lies in the supposed relationship between extracted image parameters and tumor molecular phenotype and/or genotype. It is known that genotypic heterogeneity contributes to divergent tumor biological behavior, including poor treatment response and a more aggressive phenotype. Therefore, there is growing interest in using imaging radiomic signatures either alone or in combination with other clinical or ‐omics data, eg, radiogenomics, to improve tumor phenotyping (prognostication), to allow tumor subregions with different biological characteristics that may contribute to treatment resistance to be identified/segmented for therapies, and for the prediction and evaluation of therapies. Radiomic studies have used a number of techniques including statistical methods (histogram; gray‐level co‐occurrence matrix [GLCM]; gray‐level difference matrix [GLDM], run length matrix [RLM], gray level size zone matrix [GLSZM], and neighborhood gray tone difference matrix [NGTDM]) with or without Gaussian or Wavelet transformation; and fractal‐based methods across different sequences including *T*_2_‐weighted, diffusion‐weighted, and DCE sequences. Initial radiogenomic studies including MRI have been performed in breast cancer^108^, ^109^, ^110^ renal cell carcinoma^111^ and glioma.^112^, ^113^ Variable reproducibility has been shown across different classes of features^114^ and further validation work is still required for radiomic biomarkers.

## Conclusion

- Precision cancer medicine remains a desirable goal for cancer care.
- MRI offers many advantages as a diagnostic, prognostic, predictive, or response biomarker in cancer given its capability of multiple contrast and multiparametric quantitative imaging.
- A key challenge remains to improve the efficiency of biomarker translation from discovery to implementation. Clinical translation for emerging biomarkers remains slow.
- To overcome issues regarding biomarker measurement variability across devices and across manufacturers, phantoms for quality assurance, standardization of protocols and availability of reference value databases has helped to facilitate this, alongside networks and alliances including the Quantitative Imaging Network (QIN) (http://imaging.cancer.gov/informatics/qin), the Quantitative Imaging Biomarker Alliance (QIBA) (http://www.rsna.org/qiba/); the Quantitative Imaging in Cancer: Connecting Cellular Processes to Therapy (QuIC‐ConCePT) (http://www.quic‐concept.eu/) consortium; and the American College of Radiology Imaging Network (ACRIN).
- With emerging machine‐learning approaches, quantitative MRI biomarkers will no doubt continue to expand to meet new challenges in the personalized care of oncology patients.
